# 3,3,6,6-Tetra­methyl-9-(1-methyl-1*H*-indol-2-yl)-1,2,3,4,5,6,7,8,9,10-deca­hydro­acridine-1,8-dione

**DOI:** 10.1107/S1600536812045722

**Published:** 2012-11-17

**Authors:** Sema Öztürk Yildirim, Ray J. Butcher, Ahmed El-Khouly, Cihat Safak, Rahime Şimsek

**Affiliations:** a Department of Chemistry, Howard University, 525 College Street NW, Washington, DC 20059, USA; b Department of Physics, Faculty of Sciences, Erciyes University, 38039 Kayseri, Turkey; c Hacettepe University, Faculty of Pharmacy, Dept. of Pharmaceutical Chemistry, 06100 Sihhiye-Ankara, Turkey

## Abstract

In the acridine system of the title mol­ecule, C_26_H_30_N_2_O_2_, both cyclo­hex-2-enone rings adopt sofa conformations. The indole ring system is essentially planar, with a maximum deviation of 0.017 (2) Å for a bridgehead C atom. An intra­molecular C—H⋯O hydrogen bond occurs. The mol­ecules assemble into *C*(6) chains in the crystal by way of N—H⋯O hydrogen bonds.

## Related literature

For potassium channel modulator activity for bicyclo (quinoline) and tricyclo (acridine) analogs, see: Horiuchi *et al.* (2001 ▶); Crestanello *et al.* (2000 ▶); Frank *et al.* (1993 ▶); Berkan *et al.* (2002 ▶); Şimşek *et al.* (2004 ▶); Fincan *et al.* (2012 ▶); Gündüz *et al.* (2009 ▶); Li *et al.* (2011 ▶). For a description of the Cambridge Structural Database, see: Allen (2002 ▶). For a similar structure, see: El-Khouly *et al.* (2012 ▶). For geometric analysis, see: Cremer & Pople (1975 ▶). For hydrogen-bond motifs, see: Etter *et al.* (1990 ▶).
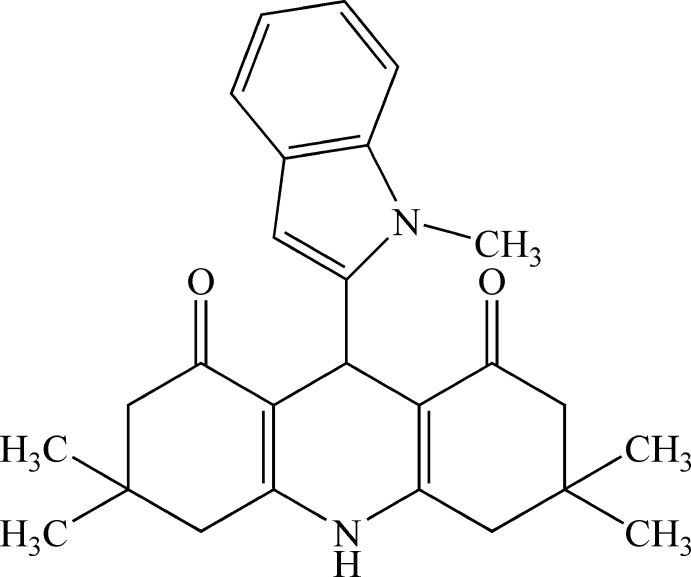


## Experimental

### 

#### Crystal data

C_26_H_30_N_2_O_2_
*M* *_r_* = 402.54
Orthorhombic, 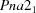

*a* = 14.09072 (13) Å
*b* = 15.04800 (15) Å
*c* = 10.39178 (12) Å
*V* = 2203.44 (4) Å^3^
*Z* = 4
Cu *K*α radiation
μ = 0.60 mm^−1^
*T* = 123 K
0.50 × 0.45 × 0.40 mm

#### Data collection

Agilent Xcalibur (Ruby, Gemini) diffractometer
Absorption correction: multi-scan [*CrysAlis RED* (Agilent, 2011 ▶), based on expressions derived from Clark & Reid (1995 ▶)] *T*_min_ = 0.753, *T*_max_ = 0.795
10170 measured reflections
3713 independent reflections
3682 reflections with *I* > 2σ(*I*)
*R*_int_ = 0.021

#### Refinement

*R*[*F* ^2^ > 2σ(*F* ^2^)] = 0.036
*wR*(*F* ^2^) = 0.096
*S* = 1.02
3713 reflections
276 parameters
1 restraint
H-atom parameters constrained
Δρ_max_ = 0.21 e Å^−3^
Δρ_min_ = −0.24 e Å^−3^

### 

Data collection: *CrysAlis PRO* (Agilent, 2011 ▶); cell refinement: *CrysAlis PRO*; data reduction: *CrysAlis PRO*; program(s) used to solve structure: *SHELXS97* (Sheldrick, 2008 ▶); program(s) used to refine structure: *SHELXL97* (Sheldrick, 2008 ▶); molecular graphics: *SHELXTL* (Sheldrick, 2008 ▶); software used to prepare material for publication: *SHELXTL*.

## Supplementary Material

Crystal structure: contains datablock(s) I, global. DOI: 10.1107/S1600536812045722/jj2157sup1.cif

Structure factors: contains datablock(s) I. DOI: 10.1107/S1600536812045722/jj2157Isup2.hkl

Supplementary material file. DOI: 10.1107/S1600536812045722/jj2157Isup3.cml

Additional supplementary materials:  crystallographic information; 3D view; checkCIF report

## Figures and Tables

**Table 1:** Hydrogen-bond geometry (Å, °)

*D*—H⋯*A*	*D*—H	H⋯*A*	*D*⋯*A*	*D*—H⋯*A*
C22—H22*C*⋯O2	0.98	2.54	3.314 (2)	136
N1—H1*A*⋯O1^i^	0.88	1.87	2.7437 (15)	170

Symmetry code: (i) 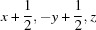.

**Fig. 1.**
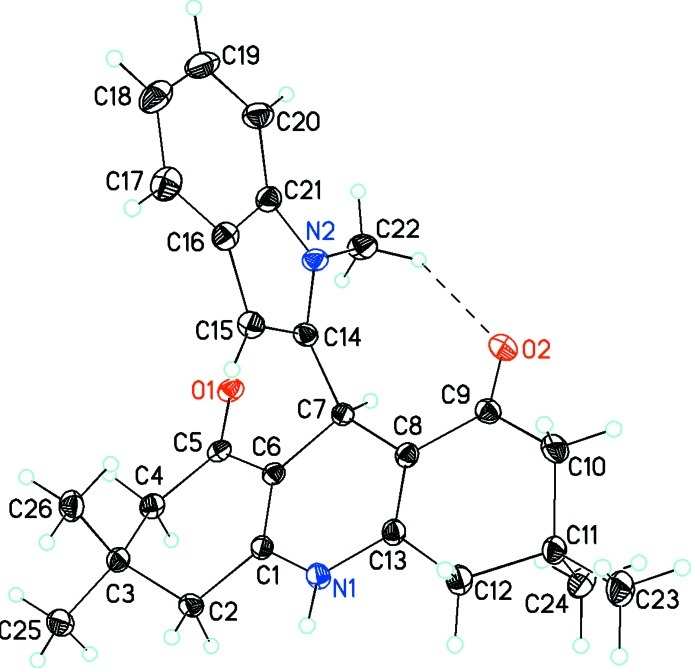
The molecular structure of the title compound showing 30% probability displacement ellipsoids for non-H atoms. Intramolecular hydrogen bond is shown as a dashed line.

**Fig. 2.**
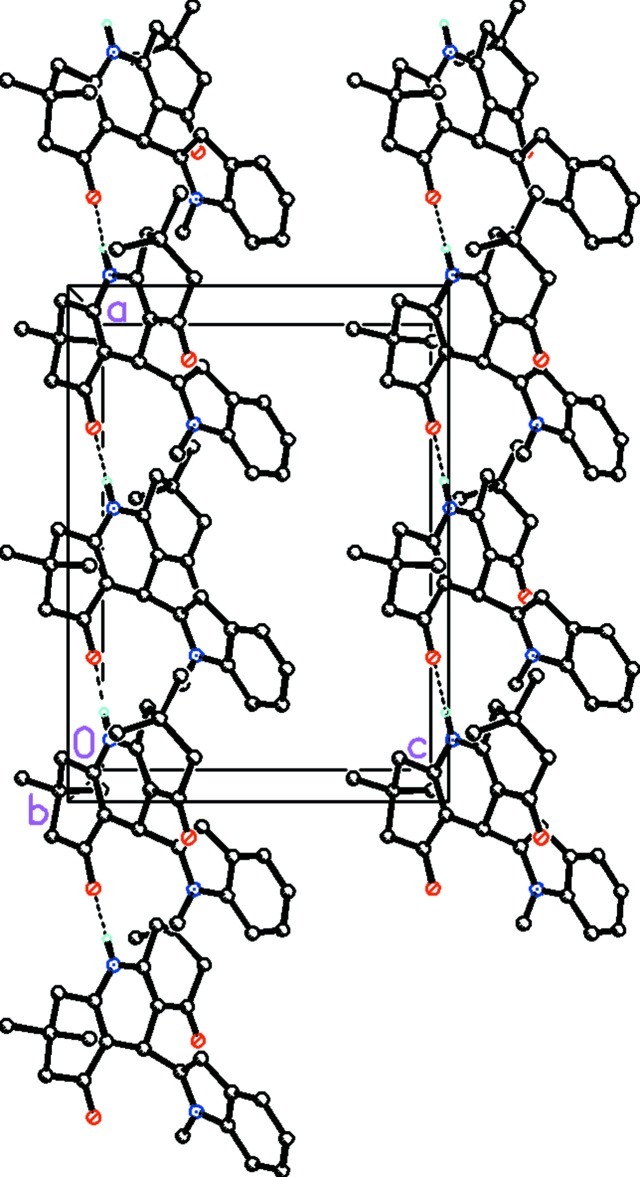
The crystal structure of the title compound, viewed along the b axis. H atoms not involved in hydrogen bonds (dashed lines) have been omitted for clarity.

## supplementary crystallographic information

### Comment

It is well known that ion channels play an important role in cell function. Potassium channels are one type of channel that regulate function in both excitable and nonexcitable cells. Potassium channel openers have the potential to restrain or prevent contractile responses of smooth muscle to excitatory stimuli. The main vasorelaxant mechanism of these openers is to increase the potassium efflux through opening plasmalemmal potassium channels, which repolarize and/or hyperpolarize the membrane. In addition to 1,4-dihydropyridine derivatives, bicyclo (quinoline) and tricyclo (acridine) analogs have also potassium channel modulator activity (Horiuchi *et al.*, 2001; Crestanello *et al.*, 2000; Frank *et al.*, 1993; Berkan *et al.*, 2002; Şimşek *et al.*, 2004; Fincan *et al.*, 2012; Gündüz *et al.*, 2009; Li *et al.*, 2011). The structure determination of the title compound, (I), was undertaken as part of our study of to 1,4-dihydropyridine derivatives.

The molecular structure of the title compound is shown in Fig. 1. Both (C1—C6 and C8–13) cyclohexene rings are in a sofa conformation with puckering parameters (Cremer & Pople, 1975) of Q_T_ = 0.464 (2) Å, θ = 54.3 (2) °, φ = 123.3 (2) ° and Q_T_ = 0.462 (2) Å, θ = 50.1 (2) °, φ = 169.5 (3) °, respectively. The 1-H indole ring (N2/C14—C21) is essentialy planar with a maximum deviation of 0.017 (2) Å for C16, and forms a dihedral angle of 81.47 (6) ° with the 1,4-dihydro-pyridine ring (N1/C1/C6—C8/C13). The bond lengths (Allen, 2002) and angles are similar to those for reported structures (El-Khouly *et al.*, 2012).

In the crystal structure, adjacent molecules interact by way of an N—H···O hydrogen bond (Fig. 2, Table 1). This results in C(6) chains (Etter, *et al.*, 1990) propagating along [010].

### Experimental

A mixture of 1-methylindole-2-carbaldehyde (1.0 mmol), 5,5-dimethyl-1,3-cyclohexanedione (2.0 mmol), ammonium acetate (5.0 mmol) was dissolved in 5 ml of methanol and refluxed until the reaction was completed (monitored by TLC). The precipitate which formed was filtered off and crystallized from ethanol. Crystals were grown by slow evaporation of a methanol solution.

### Refinement

All H atoms were positioned geometrically and refined using a riding model with C—H = 0.95–1.00 Å; N—H = 0.88 Å and *U*_iso_(H) = 1.2 *U*_eq_(C, N) or 1.5 *U*_eq_(C_methyl_). A rotating-group model was applied for the methyl groups.

### Crystal data

**Table:**

C_26_H_30_N_2_O_2_	*F*(000) = 864
*M**_r_* = 402.54	*D*_x_ = 1.213 Mg m^−^^3^
Orthorhombic, *P**n**a*2_1_	Cu *K*α radiation, λ = 1.54178 Å
Hall symbol: P 2c -2n	Cell parameters from 8837 reflections
*a* = 14.09072 (13) Å	θ = 2.9–75.6°
*b* = 15.04800 (15) Å	µ = 0.60 mm^−^^1^
*c* = 10.39178 (12) Å	*T* = 123 K
*V* = 2203.44 (4) Å^3^	Block, colorless
*Z* = 4	0.50 × 0.45 × 0.40 mm

### Data collection

**Table:**

Agilent Xcalibur (Ruby, Gemini) diffractometer	3713 independent reflections
Radiation source: Enhance (Cu) X-ray Source	3682 reflections with *I* > 2σ(*I*)
Graphite monochromator	*R*_int_ = 0.021
Detector resolution: 10.5081 pixels mm^-1^	θ_max_ = 75.7°, θ_min_ = 4.3°
ω scans	*h* = −17→17
Absorption correction: multi-scan [*CrysAlis RED* (Agilent, 2011), based on expressions derived from Clark & Reid (1995)]	*k* = −16→18
*T*_min_ = 0.753, *T*_max_ = 0.795	*l* = −13→8
10170 measured reflections	

### Refinement

**Table:**

Refinement on *F*^2^	Secondary atom site location: difference Fourier map
Least-squares matrix: full	Hydrogen site location: inferred from neighbouring sites
*R*[*F*^2^ > 2σ(*F*^2^)] = 0.036	H-atom parameters constrained
*wR*(*F*^2^) = 0.096	*w* = 1/[σ^2^(*F*_o_^2^) + (0.0699*P*)^2^ + 0.2623*P*] where *P* = (*F*_o_^2^ + 2*F*_c_^2^)/3
*S* = 1.02	(Δ/σ)_max_ = 0.001
3713 reflections	Δρ_max_ = 0.21 e Å^−^^3^
276 parameters	Δρ_min_ = −0.24 e Å^−^^3^
1 restraint	Absolute structure: Flack (1983), 1302 Friedel pairs
Primary atom site location: structure-invariant direct methods	Flack parameter: 0.17 (19)

### Special details

**Table:**

Experimental. Absorption correction: CrysAlis RED, (Agilent, 2011) Empirical absorption correction using spherical harmonics, implemented in SCALE3 ABSPACK scaling algorithm. (Clark & Reid, 1995).
Geometry. All e.s.d.'s (except the e.s.d. in the dihedral angle between two l.s. planes) are estimated using the full covariance matrix. The cell e.s.d.'s are taken into account individually in the estimation of e.s.d.'s in distances, angles and torsion angles; correlations between e.s.d.'s in cell parameters are only used when they are defined by crystal symmetry. An approximate (isotropic) treatment of cell e.s.d.'s is used for estimating e.s.d.'s involving l.s. planes.
Refinement. Refinement of *F*^2^ against ALL reflections. The weighted *R*-factor *wR* and goodness of fit *S* are based on *F*^2^, conventional *R*-factors *R* are based on *F*, with *F* set to zero for negative *F*^2^. The threshold expression of *F*^2^ > σ(*F*^2^) is used only for calculating *R*-factors(gt) *etc*. and is not relevant to the choice of reflections for refinement. *R*-factors based on *F*^2^ are statistically about twice as large as those based on *F*, and *R*- factors based on ALL data will be even larger.

### Fractional atomic coordinates and isotropic or equivalent isotropic displacement parameters (Å^2^)

**Table:**

	*x*	*y*	*z*	*U*_iso_*/*U*_eq_	
O1	0.75903 (7)	0.25747 (7)	−0.00557 (13)	0.0295 (3)	
O2	0.88973 (8)	0.50670 (7)	0.28897 (12)	0.0327 (3)	
N1	1.08387 (8)	0.31288 (8)	0.04851 (15)	0.0286 (3)	
H1A	1.1423	0.2968	0.0295	0.034*	
N2	0.76168 (8)	0.31668 (8)	0.29367 (15)	0.0277 (3)	
C1	1.01031 (10)	0.26736 (8)	−0.00433 (16)	0.0237 (3)	
C2	1.03649 (10)	0.19916 (9)	−0.10381 (18)	0.0282 (3)	
H2A	1.0974	0.1711	−0.0791	0.034*	
H2B	1.0460	0.2292	−0.1876	0.034*	
C3	0.96102 (10)	0.12665 (9)	−0.11949 (17)	0.0265 (3)	
C4	0.86446 (10)	0.17241 (10)	−0.13694 (17)	0.0284 (3)	
H4A	0.8635	0.2026	−0.2216	0.034*	
H4B	0.8141	0.1265	−0.1375	0.034*	
C5	0.84223 (10)	0.23979 (9)	−0.03302 (16)	0.0236 (3)	
C6	0.91909 (10)	0.28578 (9)	0.02966 (15)	0.0222 (3)	
C7	0.89455 (9)	0.35180 (9)	0.13511 (15)	0.0227 (3)	
H7A	0.8458	0.3941	0.1013	0.027*	
C8	0.98218 (10)	0.40439 (9)	0.17348 (16)	0.0240 (3)	
C9	0.96904 (11)	0.48130 (9)	0.25718 (16)	0.0261 (3)	
C10	1.05765 (12)	0.52769 (11)	0.30610 (18)	0.0332 (3)	
H10A	1.0796	0.4973	0.3852	0.040*	
H10B	1.0413	0.5896	0.3294	0.040*	
C11	1.13923 (10)	0.52915 (9)	0.20810 (17)	0.0284 (3)	
C12	1.15872 (10)	0.43298 (10)	0.16739 (19)	0.0319 (4)	
H12A	1.2028	0.4331	0.0932	0.038*	
H12B	1.1903	0.4014	0.2392	0.038*	
C13	1.06997 (10)	0.38350 (9)	0.13094 (17)	0.0256 (3)	
C14	0.85201 (10)	0.30123 (9)	0.24773 (16)	0.0245 (3)	
C15	0.89064 (10)	0.23076 (9)	0.31221 (17)	0.0275 (3)	
H15A	0.9521	0.2065	0.2990	0.033*	
C16	0.82205 (11)	0.20026 (10)	0.40284 (17)	0.0294 (3)	
C17	0.81850 (14)	0.13028 (12)	0.49248 (19)	0.0380 (4)	
H17A	0.8712	0.0916	0.5031	0.046*	
C18	0.73717 (15)	0.11860 (13)	0.5648 (2)	0.0441 (4)	
H18A	0.7344	0.0715	0.6256	0.053*	
C19	0.65921 (14)	0.17453 (13)	0.5502 (2)	0.0436 (4)	
H19A	0.6044	0.1648	0.6014	0.052*	
C20	0.65970 (13)	0.24415 (12)	0.4624 (2)	0.0385 (4)	
H20A	0.6064	0.2822	0.4527	0.046*	
C21	0.74192 (11)	0.25582 (10)	0.38884 (18)	0.0290 (3)	
C22	0.69626 (11)	0.38452 (11)	0.2494 (2)	0.0374 (4)	
H22A	0.6359	0.3787	0.2955	0.056*	
H22B	0.6853	0.3773	0.1568	0.056*	
H22C	0.7234	0.4434	0.2659	0.056*	
C23	1.22930 (12)	0.56706 (11)	0.2695 (2)	0.0402 (4)	
H23A	1.2813	0.5651	0.2070	0.060*	
H23B	1.2462	0.5316	0.3451	0.060*	
H23C	1.2180	0.6287	0.2956	0.060*	
C24	1.11188 (12)	0.58625 (11)	0.09208 (19)	0.0368 (4)	
H24A	1.1637	0.5857	0.0292	0.055*	
H24B	1.1002	0.6474	0.1204	0.055*	
H24D	1.0542	0.5622	0.0524	0.055*	
C25	0.98527 (11)	0.07126 (11)	−0.2383 (2)	0.0358 (4)	
H25A	0.9907	0.1103	−0.3133	0.054*	
H25D	0.9350	0.0275	−0.2533	0.054*	
H25B	1.0457	0.0404	−0.2243	0.054*	
C26	0.95850 (13)	0.06656 (10)	−0.00001 (19)	0.0364 (4)	
H26D	1.0207	0.0385	0.0117	0.055*	
H26A	0.9101	0.0205	−0.0116	0.055*	
H26B	0.9432	0.1023	0.0760	0.055*	

### Atomic displacement parameters (Å^2^)

**Table:**

	*U*^11^	*U*^22^	*U*^33^	*U*^12^	*U*^13^	*U*^23^
O1	0.0178 (5)	0.0333 (5)	0.0374 (7)	−0.0021 (4)	0.0009 (5)	0.0032 (5)
O2	0.0318 (6)	0.0337 (5)	0.0325 (7)	0.0063 (4)	0.0036 (5)	−0.0037 (5)
N1	0.0146 (5)	0.0261 (5)	0.0450 (9)	0.0008 (4)	0.0005 (6)	−0.0079 (5)
N2	0.0213 (5)	0.0287 (6)	0.0329 (8)	0.0027 (5)	0.0063 (5)	0.0026 (5)
C1	0.0196 (6)	0.0204 (6)	0.0312 (8)	0.0003 (5)	0.0001 (6)	−0.0004 (6)
C2	0.0200 (6)	0.0290 (6)	0.0355 (9)	−0.0012 (5)	0.0032 (6)	−0.0067 (6)
C3	0.0241 (6)	0.0248 (6)	0.0305 (8)	−0.0027 (5)	−0.0007 (7)	−0.0024 (6)
C4	0.0242 (6)	0.0317 (7)	0.0294 (9)	−0.0047 (5)	−0.0036 (6)	−0.0016 (6)
C5	0.0196 (6)	0.0253 (6)	0.0260 (8)	−0.0007 (5)	0.0003 (6)	0.0065 (5)
C6	0.0195 (6)	0.0227 (6)	0.0244 (8)	−0.0012 (5)	−0.0004 (5)	0.0017 (5)
C7	0.0171 (6)	0.0213 (6)	0.0297 (8)	0.0020 (4)	0.0012 (5)	0.0008 (5)
C8	0.0216 (6)	0.0221 (6)	0.0282 (8)	−0.0004 (5)	−0.0009 (6)	0.0012 (5)
C9	0.0285 (7)	0.0264 (6)	0.0235 (7)	0.0023 (5)	0.0008 (6)	0.0009 (6)
C10	0.0355 (8)	0.0356 (8)	0.0285 (9)	−0.0028 (6)	0.0002 (7)	−0.0097 (6)
C11	0.0256 (7)	0.0267 (6)	0.0328 (9)	−0.0039 (5)	−0.0021 (7)	−0.0059 (6)
C12	0.0201 (6)	0.0290 (7)	0.0467 (10)	−0.0005 (5)	−0.0030 (7)	−0.0081 (7)
C13	0.0208 (6)	0.0228 (6)	0.0333 (8)	−0.0004 (5)	−0.0017 (6)	−0.0023 (6)
C14	0.0195 (6)	0.0261 (6)	0.0279 (8)	0.0021 (5)	0.0014 (6)	−0.0025 (6)
C15	0.0251 (7)	0.0285 (7)	0.0288 (8)	0.0036 (5)	−0.0019 (6)	0.0012 (6)
C16	0.0291 (7)	0.0311 (7)	0.0279 (8)	−0.0032 (5)	−0.0033 (7)	−0.0012 (6)
C17	0.0448 (9)	0.0372 (8)	0.0321 (9)	−0.0042 (7)	−0.0083 (8)	0.0059 (7)
C18	0.0568 (11)	0.0448 (9)	0.0307 (10)	−0.0167 (8)	−0.0032 (9)	0.0093 (7)
C19	0.0450 (9)	0.0533 (10)	0.0324 (10)	−0.0181 (8)	0.0081 (8)	0.0021 (8)
C20	0.0319 (8)	0.0452 (8)	0.0383 (11)	−0.0054 (6)	0.0069 (8)	−0.0015 (8)
C21	0.0284 (7)	0.0299 (6)	0.0287 (8)	−0.0052 (5)	0.0003 (7)	−0.0010 (6)
C22	0.0256 (7)	0.0363 (7)	0.0504 (11)	0.0100 (6)	0.0095 (8)	0.0088 (7)
C23	0.0341 (8)	0.0393 (8)	0.0471 (11)	−0.0082 (7)	−0.0072 (8)	−0.0134 (8)
C24	0.0383 (8)	0.0346 (7)	0.0375 (10)	−0.0090 (6)	−0.0005 (8)	0.0030 (7)
C25	0.0306 (7)	0.0374 (8)	0.0395 (10)	−0.0054 (6)	0.0015 (8)	−0.0120 (7)
C26	0.0456 (9)	0.0249 (7)	0.0386 (10)	0.0024 (6)	0.0000 (8)	0.0028 (6)

### Geometric parameters (Å, º)

**Table:**

O1—C5	1.2356 (18)	C11—C12	1.5325 (19)
O2—C9	1.2265 (18)	C12—C13	1.5039 (18)
N1—C1	1.3583 (19)	C12—H12A	0.9900
N1—C13	1.3789 (19)	C12—H12B	0.9900
N1—H1A	0.8800	C14—C15	1.368 (2)
N2—C21	1.376 (2)	C15—C16	1.425 (2)
N2—C14	1.3790 (18)	C15—H15A	0.9500
N2—C22	1.4505 (19)	C16—C17	1.407 (2)
C1—C6	1.3614 (19)	C16—C21	1.413 (2)
C1—C2	1.503 (2)	C17—C18	1.382 (3)
C2—C3	1.5323 (18)	C17—H17A	0.9500
C2—H2A	0.9900	C18—C19	1.392 (3)
C2—H2B	0.9900	C18—H18A	0.9500
C3—C25	1.528 (2)	C19—C20	1.389 (3)
C3—C4	1.5357 (19)	C19—H19A	0.9500
C3—C26	1.536 (2)	C20—C21	1.399 (2)
C4—C5	1.514 (2)	C20—H20A	0.9500
C4—H4A	0.9900	C22—H22A	0.9800
C4—H4B	0.9900	C22—H22B	0.9800
C5—C6	1.441 (2)	C22—H22C	0.9800
C6—C7	1.519 (2)	C23—H23A	0.9800
C7—C14	1.519 (2)	C23—H23B	0.9800
C7—C8	1.5199 (18)	C23—H23C	0.9800
C7—H7A	1.0000	C24—H24A	0.9800
C8—C13	1.351 (2)	C24—H24B	0.9800
C8—C9	1.459 (2)	C24—H24D	0.9800
C9—C10	1.518 (2)	C25—H25A	0.9800
C10—C11	1.536 (2)	C25—H25D	0.9800
C10—H10A	0.9900	C25—H25B	0.9800
C10—H10B	0.9900	C26—H26D	0.9800
C11—C24	1.530 (2)	C26—H26A	0.9800
C11—C23	1.531 (2)	C26—H26B	0.9800
			
C1—N1—C13	122.10 (12)	C13—C12—H12B	109.0
C1—N1—H1A	118.9	C11—C12—H12B	109.0
C13—N1—H1A	118.9	H12A—C12—H12B	107.8
C21—N2—C14	108.87 (13)	C8—C13—N1	120.85 (13)
C21—N2—C22	124.59 (13)	C8—C13—C12	124.32 (14)
C14—N2—C22	126.53 (14)	N1—C13—C12	114.83 (12)
N1—C1—C6	120.86 (13)	C15—C14—N2	109.18 (14)
N1—C1—C2	115.80 (12)	C15—C14—C7	127.50 (13)
C6—C1—C2	123.32 (13)	N2—C14—C7	123.13 (13)
C1—C2—C3	112.90 (12)	C14—C15—C16	107.67 (13)
C1—C2—H2A	109.0	C14—C15—H15A	126.2
C3—C2—H2A	109.0	C16—C15—H15A	126.2
C1—C2—H2B	109.0	C17—C16—C21	118.88 (16)
C3—C2—H2B	109.0	C17—C16—C15	134.63 (16)
H2A—C2—H2B	107.8	C21—C16—C15	106.46 (14)
C25—C3—C2	108.61 (12)	C18—C17—C16	119.00 (17)
C25—C3—C4	110.33 (14)	C18—C17—H17A	120.5
C2—C3—C4	107.95 (11)	C16—C17—H17A	120.5
C25—C3—C26	109.69 (13)	C17—C18—C19	121.22 (17)
C2—C3—C26	110.44 (14)	C17—C18—H18A	119.4
C4—C3—C26	109.81 (13)	C19—C18—H18A	119.4
C5—C4—C3	113.54 (13)	C20—C19—C18	121.57 (17)
C5—C4—H4A	108.9	C20—C19—H19A	119.2
C3—C4—H4A	108.9	C18—C19—H19A	119.2
C5—C4—H4B	108.9	C19—C20—C21	117.24 (17)
C3—C4—H4B	108.9	C19—C20—H20A	121.4
H4A—C4—H4B	107.7	C21—C20—H20A	121.4
O1—C5—C6	120.36 (14)	N2—C21—C20	130.07 (15)
O1—C5—C4	120.34 (13)	N2—C21—C16	107.83 (13)
C6—C5—C4	119.25 (12)	C20—C21—C16	122.09 (16)
C1—C6—C5	119.64 (14)	N2—C22—H22A	109.5
C1—C6—C7	122.36 (13)	N2—C22—H22B	109.5
C5—C6—C7	117.98 (12)	H22A—C22—H22B	109.5
C6—C7—C14	108.54 (11)	N2—C22—H22C	109.5
C6—C7—C8	110.18 (11)	H22A—C22—H22C	109.5
C14—C7—C8	112.29 (13)	H22B—C22—H22C	109.5
C6—C7—H7A	108.6	C11—C23—H23A	109.5
C14—C7—H7A	108.6	C11—C23—H23B	109.5
C8—C7—H7A	108.6	H23A—C23—H23B	109.5
C13—C8—C9	119.72 (13)	C11—C23—H23C	109.5
C13—C8—C7	122.47 (13)	H23A—C23—H23C	109.5
C9—C8—C7	117.79 (12)	H23B—C23—H23C	109.5
O2—C9—C8	121.55 (14)	C11—C24—H24A	109.5
O2—C9—C10	121.06 (14)	C11—C24—H24B	109.5
C8—C9—C10	117.38 (13)	H24A—C24—H24B	109.5
C9—C10—C11	113.58 (14)	C11—C24—H24D	109.5
C9—C10—H10A	108.8	H24A—C24—H24D	109.5
C11—C10—H10A	108.8	H24B—C24—H24D	109.5
C9—C10—H10B	108.8	C3—C25—H25A	109.5
C11—C10—H10B	108.8	C3—C25—H25D	109.5
H10A—C10—H10B	107.7	H25A—C25—H25D	109.5
C24—C11—C23	109.14 (13)	C3—C25—H25B	109.5
C24—C11—C12	110.98 (14)	H25A—C25—H25B	109.5
C23—C11—C12	108.56 (12)	H25D—C25—H25B	109.5
C24—C11—C10	110.00 (13)	C3—C26—H26D	109.5
C23—C11—C10	110.47 (14)	C3—C26—H26A	109.5
C12—C11—C10	107.68 (13)	H26D—C26—H26A	109.5
C13—C12—C11	112.83 (12)	C3—C26—H26B	109.5
C13—C12—H12A	109.0	H26D—C26—H26B	109.5
C11—C12—H12A	109.0	H26A—C26—H26B	109.5
			
C13—N1—C1—C6	−4.8 (2)	C23—C11—C12—C13	168.26 (16)
C13—N1—C1—C2	173.19 (14)	C10—C11—C12—C13	48.64 (19)
N1—C1—C2—C3	156.80 (14)	C9—C8—C13—N1	−177.92 (14)
C6—C1—C2—C3	−25.2 (2)	C7—C8—C13—N1	0.6 (2)
C1—C2—C3—C25	169.70 (14)	C9—C8—C13—C12	2.1 (2)
C1—C2—C3—C4	50.08 (18)	C7—C8—C13—C12	−179.34 (15)
C1—C2—C3—C26	−69.98 (17)	C1—N1—C13—C8	7.2 (2)
C25—C3—C4—C5	−170.93 (13)	C1—N1—C13—C12	−172.86 (15)
C2—C3—C4—C5	−52.40 (18)	C11—C12—C13—C8	−24.4 (2)
C26—C3—C4—C5	68.06 (15)	C11—C12—C13—N1	155.59 (15)
C3—C4—C5—O1	−153.53 (14)	C21—N2—C14—C15	0.16 (19)
C3—C4—C5—C6	29.08 (19)	C22—N2—C14—C15	−178.97 (16)
N1—C1—C6—C5	176.66 (14)	C21—N2—C14—C7	175.40 (14)
C2—C1—C6—C5	−1.2 (2)	C22—N2—C14—C7	−3.7 (3)
N1—C1—C6—C7	−5.2 (2)	C6—C7—C14—C15	54.4 (2)
C2—C1—C6—C7	176.95 (14)	C8—C7—C14—C15	−67.67 (19)
O1—C5—C6—C1	−178.11 (14)	C6—C7—C14—N2	−119.94 (15)
C4—C5—C6—C1	−0.7 (2)	C8—C7—C14—N2	118.01 (15)
O1—C5—C6—C7	3.6 (2)	N2—C14—C15—C16	−0.04 (19)
C4—C5—C6—C7	−178.96 (13)	C7—C14—C15—C16	−175.01 (14)
C1—C6—C7—C14	−112.01 (15)	C14—C15—C16—C17	177.88 (18)
C5—C6—C7—C14	66.18 (16)	C14—C15—C16—C21	−0.09 (18)
C1—C6—C7—C8	11.32 (19)	C21—C16—C17—C18	−0.6 (3)
C5—C6—C7—C8	−170.49 (12)	C15—C16—C17—C18	−178.36 (19)
C6—C7—C8—C13	−9.1 (2)	C16—C17—C18—C19	0.1 (3)
C14—C7—C8—C13	112.07 (16)	C17—C18—C19—C20	0.1 (3)
C6—C7—C8—C9	169.54 (13)	C18—C19—C20—C21	0.1 (3)
C14—C7—C8—C9	−69.34 (16)	C14—N2—C21—C20	−179.10 (17)
C13—C8—C9—O2	173.83 (16)	C22—N2—C21—C20	0.1 (3)
C7—C8—C9—O2	−4.8 (2)	C14—N2—C21—C16	−0.22 (18)
C13—C8—C9—C10	−7.5 (2)	C22—N2—C21—C16	178.94 (16)
C7—C8—C9—C10	173.86 (14)	C19—C20—C21—N2	178.18 (18)
O2—C9—C10—C11	−145.85 (15)	C19—C20—C21—C16	−0.6 (3)
C8—C9—C10—C11	35.5 (2)	C17—C16—C21—N2	−178.16 (15)
C9—C10—C11—C24	66.05 (16)	C15—C16—C21—N2	0.19 (18)
C9—C10—C11—C23	−173.41 (13)	C17—C16—C21—C20	0.8 (3)
C9—C10—C11—C12	−55.01 (18)	C15—C16—C21—C20	179.18 (16)
C24—C11—C12—C13	−71.80 (18)		

### Hydrogen-bond geometry (Å, º)

**Table:**

*D*—H···*A*	*D*—H	H···*A*	*D*···*A*	*D*—H···*A*
C22—H22*C*···O2	0.98	2.54	3.314 (2)	136
N1—H1*A*···O1^i^	0.88	1.87	2.7437 (15)	170

Symmetry code: (i) *x*+1/2, −*y*+1/2, *z*.
